# Obstructive jaundice due to retroperitoneal desmoid-type fibromatosis: a case report and review of literature

**DOI:** 10.1097/MS9.0000000000003759

**Published:** 2025-08-28

**Authors:** Deekshya Devkota, Chandan Kumar Sah, Madhav Dulal, Sabnam Singh

**Affiliations:** a Nepalese Army Institute of Health Sciences, Kathmandu, Nepal; b Department of Pathology, Shree Birendra Hospital, Kathmandu, Nepal

**Keywords:** case report, chemotherapy, desmoid fibromatosis, obstructive jaundice

## Abstract

**Introduction and importance:**

Desmoid-type fibromatosis (DF) is a rare, benign fibroblastic tumor arising from musculoaponeurotic structures. Despite lacking metastatic potential, it can be locally aggressive. Retroperitoneal DF is uncommon, and its presentation with obstructive jaundice is extremely rare.

**Case presentation:**

A 55-year-old male with no significant medical or family history presented with a six-month history of right upper quadrant abdominal pain and three weeks of progressive jaundice. Laboratory findings revealed a cholestatic pattern and elevated CA 19-9. Imaging, histopathological examination and immunohistochemistry were consistent with desmoid-type fibromatosis. The patient was managed with palliative chemotherapy and percutaneous transhepatic biliary drainage (PTBD) to relieve biliary obstruction.

**Clinical discussion:**

Retroperitoneal DF is typically managed with surgical excision or watchful waiting, depending on the location and symptoms. While surgical resection is preferred, it is often not feasible in cases involving extensive invasion. In this case, chemotherapy served as the primary treatment modality due to the tumor’s unresectability.

**Conclusion:**

This case highlights the importance of including retroperitoneal desmoid-type fibromatosis in the differential diagnosis of obstructive jaundice and underscores the need for larger studies and updated management guidelines.

## Introduction

Desmoid-type fibromatosis (DF), also known as desmoid tumor/deep fibromatosis/aggressive fibromatosis, is a rare mesenchymal neoplasm characterized by locally aggressive fibroblastic proliferation arising in deep soft tissues^[1]^. It accounts for 0.03% of all neoplasms and <3% of all soft tissue tumors^[2]^. The local recurrence rate is high and varies from 18% to 56%^[3]^. DF is categorized by location as extra-abdominal (60%), abdominal wall (25%), and intra-abdominal (8-15%)^[4]^.

Desmoid-type fibromatosis (DF) typically occurs sporadically, but about 7.5–16% of cases are associated with familial adenomatous polyposis (FAP) and linked to mutations in the APC gene on chromosome 5q22^[5,6]^. Retroperitoneal DF is usually related to familial syndromes and only rarely arises sporadically^[7]^. Most patients with intra-abdominal tumors remain asymptomatic until the lesion grows large enough to cause symptoms involving the intestines, vasculature, ureters, or nerves, with pain being the most frequent complaint^[8]^. The biological behavior of DF varies from spontaneous regression and long-term stability to invasion of adjacent organs and neurovascular structures^[9]^.

**HIGHLIGHTS**

- This case reports an exceptionally rare presentation of retroperitoneal desmoid-type fibromatosis causing obstructive jaundice.
- Due to unresectability of the tumor, the patient was managed successfully with palliative chemotherapy and percutaneous transhepatic biliary drainage.
- Retroperitoneal DF should be included in the differential diagnosis of obstructive jaundice, especially in atypical cases.

While imaging studies can assist in the diagnosis, histopathology and immunohistochemistry remain the gold standard for confirmation^[10]^. Clinical management depends on the location and size of the tumor. In 2017, an updated guideline of the European desmoid working group stated that medical therapy should be a first-line treatment for patients with retroperitoneal desmoid-type fibromatosis (RPDF) with surgery or definitive chemoradiation as an option if further progression occurs^[11]^.

Obstructive Jaundice is commonly caused by biliary stones, strictures, or malignancies, but secondary to retroperitoneal desmoid fibromatosis is a rare occurrence. Here, we report a case of obstructive jaundice due to sporadic retroperitoneal desmoid fibromatosis.

The report adheres to the Surgical Case Report (SCARE) guidelines^[12]^ and presents the patient’s clinical presentation, investigations, management, and outcomes.

## Case presentation

A 55-year-old male with no comorbidities presented with 6 months of right upper quadrant abdominal pain and 3 weeks of jaundice. He reported clay-colored stools, dark urine, and nausea, but no pruritus, fever, or vomiting. Examination revealed icterus and right hypochondrial tenderness. Labs showed deranged liver function and elevated CA 19-9; other tests were normal (Table 1).

**Table 1:** The patient’s laboratory workup on admission

Parameter	Value	Reference range
WBC	7.1 × 10^3^/µL	(4–11) × 10^3^/µL
Hb	12.9 gm/dL	(13–17) gm/dL
PLT	254 × 10^3^	(150–450) × 10^3^/µL
Blood group	A positive	
PT	11.6s	11–13 s
INR	1.1	0.8–1.3
Serology	Non–reactive	
Total bilirubin	15.99 mg/dL	0.3–1.2 mg/dL
Direct bilirubin	12.40 mg/dL	0–0.2 mg/dL
ALT	231.2 U/L	13–40 U/L
AST	149.4 U/L	13–40 U/L
ALP	819 U/L	42–128 U/L
Urea	23.4 mg/dL	13–43 mg/dL
Creatinine	1.02 mg/dL	0.6–1.3 mg/dL
RBS	80.1 mg/dL	70–140 mg/dL
CA 19-9	154.3 U/mL	0–35 U/mL
CEA	3.55 ng/mL	<5 ng/mL
AFP	2.98 IU/mL	0–10 IU/mL

CBC, complete blood count; WBC, white blood cells; Hb, hemoglobin; PLT, platelets; PT/INR, Prothrombin time/International normalized ratio; ALT, alkaline aminotransferase; AST, aspartate aminotransferase; ALP, Alkaline phosphatase; RBS, Random Blood Sugar; CA 19-9, Carbohydrate antigen 19-9; CEA, Carcinoembryonic antigen; AFP, Alpha feto protein.

**Figure 1.**
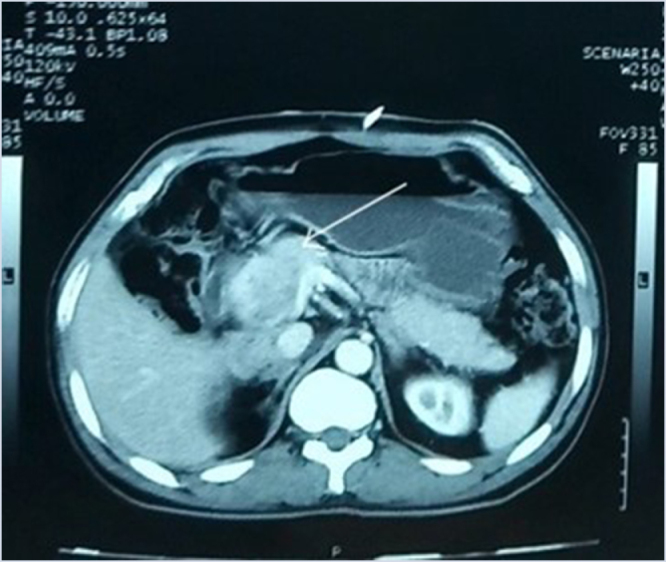
An axial section of the CECT abdomen shows a heterogeneously enhancing mass in the right retroperitoneum compressing the distal part of CBD (white arrow).

A contrast enhanced computed tomography (CECT) scan of the abdomen and pelvis revealed a heterogeneously enhancing soft density mass measuring 7.3 × 7.1 × 9.8 cm (AP × TR × CC) in the right retroperitoneum, closely abutting the uncinate process of the pancreas and the second part of the duodenum. The post-contrast study showed mild lesion enhancement with a few non-enhancing areas indicative of necrotic changes. The mass compresses the IVC, the distal part of the CBD, leading to proximal dilation of up to 13.4 mm and IHBR dilation in both liver lobes. The gallbladder is distended, measuring 10 × 4.5 cm, with slightly hyperdense content, likely sludge. Retroperitoneal lymphadenopathy and hepatic hemangioma are also observed. A follow-up CECT a month later showed an increase in the size of the mass to approximately 7.4 × 9.3 × 11.2 cm (AP × TR × CC) (Fig. 1).

**Figure 2.**
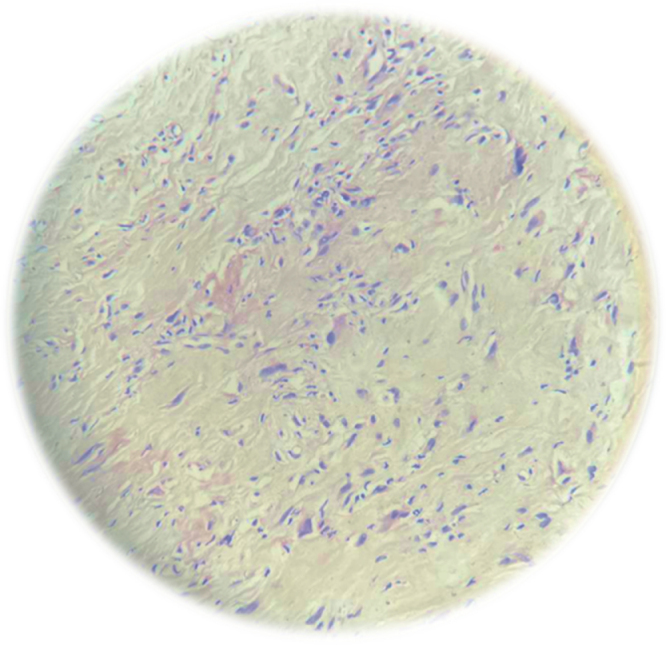
Microscopic examination reveals long, sweeping fascicles of spindle cells within a collagenous stroma. These cells exhibit uniform wavy nuclei and pale cytoplasm, without nuclear hyperchromasia, atypia, or mitosis.

He underwent percutaneous transhepatic biliary drainage (PTBD) to relieve symptoms of obstructive jaundice. Post-PTBD laboratory evaluation revealed a total bilirubin of 4.63 mg/dL, direct bilirubin of 3.80 mg/dL, and ALP of 299 U/L. An ultrasound-guided trucut biopsy of the retroperitoneal mass showed long, sweeping fascicles of spindle cells within a collagenous stroma. The cells had uniform wavy nuclei and pale cytoplasm, without nuclear hyperchromasia or atypia, consistent with desmoid-type fibromatosis (Fig. 2). He was advised to undergo immunohistochemistry at an external center for further evaluation and diagnostic confirmation.

Immunohistochemistry revealed that a few tumor cells exhibited focal nuclear positivity for β-catenin, focal positivity for SMA, and desmin positivity, as well as CD34 positivity in vessels, whereas they remained negative for CD117 and S-100 in tumor cells. This immunohistochemistry pattern is consistent with DF.

The patient received five cycles of chemotherapy with liposomal doxorubicin 60 mg and Pegylated r-human granulocyte colony-stimulating factor 6 mg, administered monthly. After completion of five cycles, a contrast-enhanced CT (CECT) of the abdomen and pelvis was performed. Compared to the previous scan, it demonstrated a slight decrease in the size of the retroperitoneal mass, now measuring 6.2 × 9.2 × 9.5 cm, with no significant change in size and extent of the retroperitoneal lymphadenopathy.

One month later, the patient presented to the emergency department with complaints of multiple episodes of vomiting, pallor, and bilateral pitting pedal edema. Ultrasound of the abdomen and pelvis revealed moderate ascites, bilateral pleural effusion, and features suggestive of cystitis, which was managed conservatively.

Given the IVC compression and no significant reduction in tumor size in order to consider surgical resection, management was continued with palliative chemotherapy and percutaneous transhepatic biliary drainage (PTBD). Due to loss to follow-up, further evaluation could not be done.

## Discussion

Fibromatosis can be categorized based on its connective tissue origin as either superficial (fascial) or deep (musculoaponeurotic)^[13]^. In 2002, the WHO defined “desmoid-type fibromatosis” as a deep, non-metastatic fibroblastic tumor, commonly known as a desmoid tumor^[14]^. Despite being benign, desmoid tumors can be locally aggressive, causing compression or obstruction of nearby organs and vessels^[15]^.

Known risk factors for DF include APC and beta-catenin (CTNNB1) gene mutations, as in familial adenomatous polyposis, along with prior surgery, trauma, pregnancy, and oral contraceptive use. However, its exact pathogenesis remains unclear^[14]^. In our patient, there were no risk factors or family history predisposing him to DF.

Patients with DF are often asymptomatic or present with nonspecific symptoms like weight loss or abdominal pain. Painless jaundice is rare in pancreatic head DF since it typically does not obstruct the bile duct; however, our patient developed obstructive jaundice from tumor compression of the common bile duct.

When feasible, retroperitoneal DF is treated by wide-margin surgical resection; however, DF may recur even with complete surgical resection^[2]^. According to the European Society of Medical Oncology (ESMO), asymptomatic DF in a non-life-threatening location can be carefully watched without active intervention^[16]^. In our case, due to the large size of the tumor compressing the IVC and common bile duct, chemotherapy was initiated to reduce the tumor size to enable surgical resection. However, there was no significant reduction in size, and the patient was subsequently managed with palliative chemotherapy alone.

*Lee et al*^[1]^ reported a case involving a 46-year-old male who presented with left upper quadrant abdominal pain and was diagnosed with retroperitoneal desmoid-type fibromatosis. He was successfully managed with a laparoscopic spleen-preserving distal pancreatectomy. *Charif et al*^[7]^ described a 53-year-old male presenting with urinary urgency, who was diagnosed with retroperitoneal DF causing left-sided hydroureteronephrosis due to tumor-related obstruction. The patient was managed with surgical excision.

Another case report by *Fathia et al*^[9]^ describes a 31-year-old woman with a history of familial adenomatous polyposis, who was diagnosed with retroperitoneal DF infiltrating the inferior duodenal flexure. She was managed with a total colectomy with ileoanal anastomosis and excision of the retroperitoneal mass.

In most previously reported cases, DF has been managed either through surgical excision or a watchful waiting approach. However, we present a unique case in which the tumor was not amenable to resection and was therefore managed with palliative chemotherapy with pegylated liposomal doxorubicin and PTBD for obstructive jaundice. A clinical trial conducted by *Constantinidou et al*^[17]^ involving 12 patients demonstrated that pegylated liposomal doxorubicin, used as a single-agent chemotherapy, exhibits promising activity with acceptable toxicity and may even offer long-term benefits in cases of unresectable desmoid fibromatosis.

A key limitation of this case report is the inability to obtain long-term outcome due to patient loss to follow-up.

## Conclusion

Given the rarity of desmoid-type fibromatosis in the retroperitoneum and its role in causing obstructive jaundice as in this case, this case report emphasizes the importance of considering retroperitoneal desmoid fibromatosis in the differential diagnosis of obstructive jaundice. It also highlights the need for large-scale trials and updated guidelines for its management.

## Data Availability

Available on request. A copy of the written consent is available for review by the editorial office of this journal.

## Research registration unique identifying number (UIN)

Not applicable to case report.
